# Innovative Diagnostic and Therapeutic Interventions in Cervical Dysplasia: A Systematic Review of Controlled Trials

**DOI:** 10.3390/cancers14112670

**Published:** 2022-05-27

**Authors:** Julia M. Hecken, Günther A. Rezniczek, Clemens B. Tempfer

**Affiliations:** 1 Department of Gynecology and Obstetrics, Ruhr-Universität Bochum, Marien Hospital Herne, 44625 Herne, Germany; julia.hecken@rub.de (J.M.H.); guenther.rezniczek@rub.de (G.A.R.); 2 Ruhr-Universität Comprehensive Cancer Center (RUCCC), Ruhr-Universität Bochum, 44801 Bochum, Germany

**Keywords:** cervical dysplasia, colposcopy, conization, human papilloma virus

## Abstract

**Simple Summary:**

Cervical dysplasia is one of the most common disorders of the female genital tract affecting millions of women worldwide. This systematic review of the literature of the last decade shows that significant progress has been made in its diagnosis and treatment. Based on >30 controlled clinical trials, specific and evidence-based recommendations can be formulated, such as for intravenous or intracervical lidocaine for pain reduction during colposcopically-directed cervical biopsies, but not topical lidocaine, music, or video colposcopy. Monsel’s solution might be used to control bleeding after cervical biopsies. The acetic acid test should be scored after 1 min and should be followed by Lugol’s iodine test for an optimal detection of dysplastic lesions. Loop electrical excision using standard instrumentation and techniques should be performed under local anesthesia and with direct colposcopic vision. Cryotherapy and thermoablation might be used in women with low-grade dysplasia, especially in women with HIV infection. Topical imiquimod remains an experimental procedure.

**Abstract:**

Cervical dysplasia is a common precancerous lesion affecting 1% to 2% of women worldwide. Significant progress in the diagnosis and treatment of cervical dysplasia have been made in the last decade. We performed a systematic literature search of the databases PubMed and Cochrane Central Register of Controlled Trials to identify controlled clinical trials reporting on the efficacy and safety of diagnostic and therapeutic interventions for cervical dysplasia. Data were analyzed according to PRISMA guidelines. In total, 33 studies reporting on 5935 women were identified. We recommend intravenous or intracervical lidocaine for pain reduction during colposcopically-directed cervical biopsies but not topical lidocaine, music, or video colposcopy. Monsel’s solution might be used to control bleeding after cervical biopsies. The acetic acid test should be scored 1 min after the application of acetic acid and should be followed by Lugol’s iodine test for an optimal yield of LSIL/HSIL. LEEP/LLETZ remains the standard and techniques such as SWETZ, C-LETZ, and TCBEE are not superior. LEEP/LLETZ should be performed under local anesthesia and with direct colposcopic vision. Cryotherapy and thermoablation might be used in women with LSIL, especially in women with HIV infection, but LEEP/LLETZ remains the standard for HSIL. Topical imiquimod remains an experimental procedure. In conclusion, significant progress has been made in the last decade regarding both diagnostic interventions as well as therapeutic interventions for women with cervical dysplasia. Based on >30 controlled clinical trials, we were able to formulate specific and evidence-based recommendations.

## 1. Introduction

Cervical dysplasia is one of the most common disorders in gynecology. Specifically, precancerous lesions of the cervix such as low-grade squamous intraepithelial lesions (LSIL) and high-grade squamous intraepithelial lesions (HSIL) affect 1% to 3% of women taking part in national cervical cancer screening programs [1,2]. Cervical dysplasia is a virus-associated disorder and is caused in >95% of cases by an infection with high-risk subtypes of the Human Papilloma Virus (HPV), which ranks among the most common sexually transmitted infectious diseases worldwide [3]. Subsequent to the high incidence of cervical dysplasia, surgical interventions aimed at treating LSIL/HSIL before they develop into invasive cervical cancer are among the most common surgical interventions in Gynecology. For example, approximately half a million loop electrosurgical excision (LEEP) and large loop excision of the transformation zone (LLETZ) procedures are being performed in the United States each year [4]. Aside from the immediate consequences of cervical surgery such as pain, anxiety, and costs, LEEP/LLETZ also causes long-term adverse effects such as an increased risk of preterm delivery. Specifically, the height of the removed cone [5] and the presence of an HPV-related lesion alone, even without the execution of an excisional treatment [6], has been associated with a worsening of obstetric outcomes. In a systematic review of 32 studies, for example, Monti et al. found a significantly elevated risk of premature delivery, low birth weight, and premature rupture of membranes in women with a history of surgery for cervical dysplasia [7]. This statistically and clinically significant increase in obstetrical risks is directly correlated with the number and extent of cervical procedures such as LEEP and LLETZ [8]. Therefore, effective means for the management of cervical dysplasia are a major medical need for women worldwide. Evidence-based strategies for the diagnosis and treatment of cervical dysplasia are necessary to guarantee optimal outcomes and to avoid or minimize long-term sequelae, such as premature birth. In the last decade, numerous randomized controlled trials (RCTs) and non-randomized prospective controlled trials (PCTs) have been performed designed to refine the diagnosis of LSIL/HSIL with interventions such as colposcopy, acetic acid test, and Lugol’s iodine test. Likewise, numerous RCTs and PCTs have been performed aimed at improving the efficacy and safety of surgical interventions such as cryotherapy, LEEP, and LLETZ. In the present systematic review, we summarize all RCTs and PCTs assessing diagnostic and therapeutic interventions for cervical dysplasia published during the last decade. Based on the results of these studies, we comprehensively discuss the current evidence-based standard of care for the management of women with cervical dysplasia.

## 2. Materials and Methods

**Figure 1:**
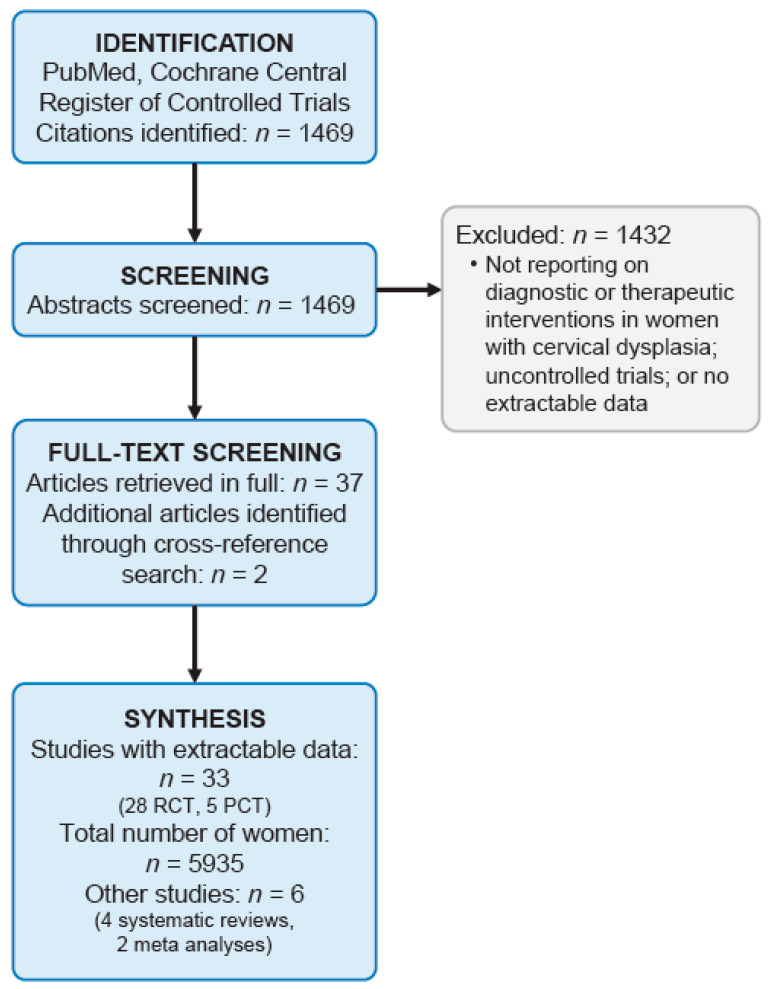
Flow diagram of the literature search algorithm.

We performed a systematic literature search of the databases PubMed and Cochrane Central Register of Controlled Trials using the search terms ((“uterine cervical dysplasia”[MeSH Terms] OR (“uterine”[All Fields] AND “cervical”[All Fields] AND “dysplasia”[All Fields]) OR “uterine cervical dysplasia”[All Fields] OR (“cervical”[All Fields] AND “dysplasia”[All Fields]) OR “cervical dysplasia”[All Fields] OR “cervical intraepithelial neoplasia”[MeSH Terms] OR (“cervical”[All Fields] AND “intraepithelial”[All Fields] AND “neoplasia”[All Fields]) OR “cervical intraepithelial neoplasia”[All Fields] OR (“cervical”[All Fields] AND “dysplasia”[All Fields])) AND (“colposcopy”[MeSH Terms] OR “colposcopy”[All Fields] OR “colposcopies”[All Fields])) OR ((“conisation”[All Fields] OR “conization”[MeSH Terms] OR “conization”[All Fields] OR “conisations”[All Fields] OR “conizations”[All Fields] OR “conized”[All Fields]) AND (“random allocation”[MeSH Terms] OR (“random”[All Fields] AND “allocation”[All Fields]) OR “random allocation”[All Fields] OR “random”[All Fields] OR “randomization”[All Fields] OR “randomized”[All Fields] OR “randomisation”[All Fields] OR “randomisations”[All Fields] OR “randomise”[All Fields] OR “randomised”[All Fields] OR “randomising”[All Fields] OR “randomizations”[All Fields] OR “randomize”[All Fields] OR “randomizes”[All Fields] OR “randomizing”[All Fields] OR “randomness”[All Fields] OR “randoms”[All Fields])) (search date: 15 March 2022). The methodology followed the Preferred Reporting Items for Systematic Reviews and Meta-Analyses (PRISMA) criteria [9]. The Population/Problem–Intervention/Exposure–Comparison–Outcome (PICO) question [10] defined to guide the selection of studies was as follows: What are the optimal diagnostic and therapeutic procedures for women with cervical dysplasia based on controlled trials with regard to diagnostic sensitivity and specificity, treatment efficacy, and side effects? Screening, eligibility, and data analysis were performed by two authors independently (JMH and CBT). Discrepancies were solved by consensus. Study investigators were not contacted to obtain further information. The literature search was restricted to controlled trials, i.e., RCTs and PCTs, defined as prospective cohort studies with upfront-defined inclusion/exclusion criteria and outcomes. Methodological quality was assessed in all studies using the Cochrane RoB 2.0 (randomized trials; [11]) or MINORS (non-randomized trials; [12]) tools. with the above-described search strategy, we identified 5076 citations. Therefore, the search was restricted to the last 10 y, i.e., published January 2012 or later. After screening all abstracts, appropriate citations, i.e., those reporting on diagnostic and therapeutic interventions in women with cervical dysplasia within a controlled trial setting, were selected. Studies not reporting individual patient data, uncontrolled trials, and studies containing no extractable clinical data were excluded. All citations were then retrieved in full and cross reference searching was performed in order to identify further studies. Figure 1 shows a flow diagram of the literature search algorithm. Data were extracted and analyzed in a descriptive manner. Meta-analysis was not performed due to the heterogeneity of studies. The protocol for this review has not been registered.

In order to identify ongoing clinical trials, we additionally searched the website of the National Institutes of Health clinical trials database (www.clinicaltrials.gov) using the search term “cervical dysplasia” (search date: 4 April 2022). We selected only studies assessing diagnostic and therapeutic interventions in women with proven or suspected cervical dysplasia. Studies evaluating screening strategies for cervical dysplasia were not included.

## 3. Results

In a systematic literature search using the search criteria as described above (search date: 15 March 2022), we identified 1469 citations. 1432 citations were excluded because they did not report on diagnostic and therapeutic interventions in women with cervical dysplasia within a controlled trial setting as defined for the purpose of this review. Using the remaining 37 citations, cross reference searching identified two further appropriate citations. Thus, in summary, 39 citations reporting on diagnostic and therapeutic interventions in women with cervical dysplasia within a controlled trial setting were included in this review [13,14,15,16,17,18,19,20,21,22,23,24,25,26,27,28,29,30,31,32,33,34,35,36,37,38,39,40,41,42,43,44,45,46,47,48,49,50,51]. Among them, we found 5 PCTs [13,14,15,16,17] and 28 RCTs [18,19,20,21,22,23,24,25,26,27,28,29,30,31,32,33,34,35,36,37,38,39,40,41,42,43,44,45], describing in summary 5935 patients. In addition, we found 6 systematic reviews and meta-analyses [46,47,48,49,50,51].

The clinical characteristics of the 33 studies reporting individual patient data are shown in Table 1 and Table 2. Specifically, the clinical characteristics of individual studies reporting on diagnostic procedures in women with cervical dysplasia are shown in Table 1, and those reporting on therapeutic procedures in women with cervical dysplasia are shown in Table 2. Table 3 shows the clinical characteristics of 27 ongoing studies assessing diagnostic and therapeutic interventions in women with cervical dysplasia listed within the National Institutes of Health clinical trials database (www.clinicaltrials.gov, search date: 4 April 2022).

### 3.1. Diagnostic Studies in Women with Suspected or Proven Cervical Dysplasia

The clinical characteristics of individual studies reporting on diagnostic procedures in women with cervical dysplasia are shown in Table 1. We identified 12 studies. In total, 8 studies with 1390 participants were RCTs [18,19,20,21,22,23,24,25] and 4 studies with 893 participants were PCTs [13,14,15,16]. In 7 RCTs, interventions aimed at reducing pain during colposcopy and colposcopically-controlled cervical biopsies were evaluated [18,20,21,22,23,24,25]. In summary, these studies demonstrate that intravenous or intracervical lidocaine is efficacious for reducing pain [21,24,25] (this was not seen in one PCT [13]), whereas mixed results were reported for topical lidocaine spray on the cervix vs. placebo or forced coughing [18,23]. In addition, music as well as video colposcopy (with the patient watching the procedure) did not reduce pain during colposcopy [20,22]. One RCT looked at bleeding control after colposcopically-controlled biopsies by use of Monsel’s solution demonstrating that Monsel’s solution was efficacious in reducing blood loss and duration after biopsies [19]. Two PCTs evaluated the optimal use of the acetowhite acid test [14] and Lugol’s iodine test [16]. The best time to identify acetowhite lesions was 1 min after the application of acetic acid with fading of acetowhite lesions being common and time-dependent supporting a recommendation of not prolonging colposcopy beyond 3 min [14]. Lugol’s iodine showed moderate sensitivity and poor specificity, but it changed the clinical management in 5% of cases when used in addition to acetic acid [16]. Finally, one PCT found that 4 random cervical biopsies at the squamocolumnar junction resulted in an optimal yield of cervical intraepithelial neoplasia (CIN) 2+ lesions in women with a cytology of LSIL or Atypical Squamous Cells of Undetermined Significance (ASCUS) who had a normal colposcopic impression [15].

### 3.2. Therapeutic Studies in Women with Suspected or Proven Cervical Dysplasia

The clinical characteristics of individual studies reporting on therapeutic procedures in women with cervical dysplasia are shown in Table 2. We identified 20 RCTs with 3355 participants [26,27,28,29,30,31,32,33,34,35,36,37,38,39,40,41,42,43,44,45] and one PCT with 297 participants [17]. in total, 4 RCTs found that alternative electrosurgical techniques such as Straight Wire Excision of the Transformation Zone (SWETZ), Contour-Loop Excision of the Transformation Zone (C-LETZ), and True Cone Biopsy Electrode Excision (TCBEE) were comparable to the standard LEEP/LLETZ procedure with minimal differences regarding specimen fragmentation and endocervical resection margin status [26,27,28,31]. LEEP performed under direct colposcopic vision led to smaller cone sizes without compromising margin status [33], but video colposcopy did not have this benefit [44]. Spray coagulation was better than forced coagulation for intra-operative bleeding control [29] and a chitosan tampon effectively reduced post-operative bleeding episodes [37]. Patients preferred LEEP under local anesthesia over general anesthesia [38]. Cryotherapy as well as thermoablation were found to be safe and efficacious in women with LSIL, especially in women with HIV infection [30,32,36,39], but LLETZ was superior when treating HSIL [34]. Topical imiquimod was efficacious for the treatment of LSIL/HSIL but was less effective than surgery [40,45].

### 3.3. Methodological Assessment of Diagnostic and Therapeutic Studies in Women with Cervical Dysplasia

**Figure 2:**
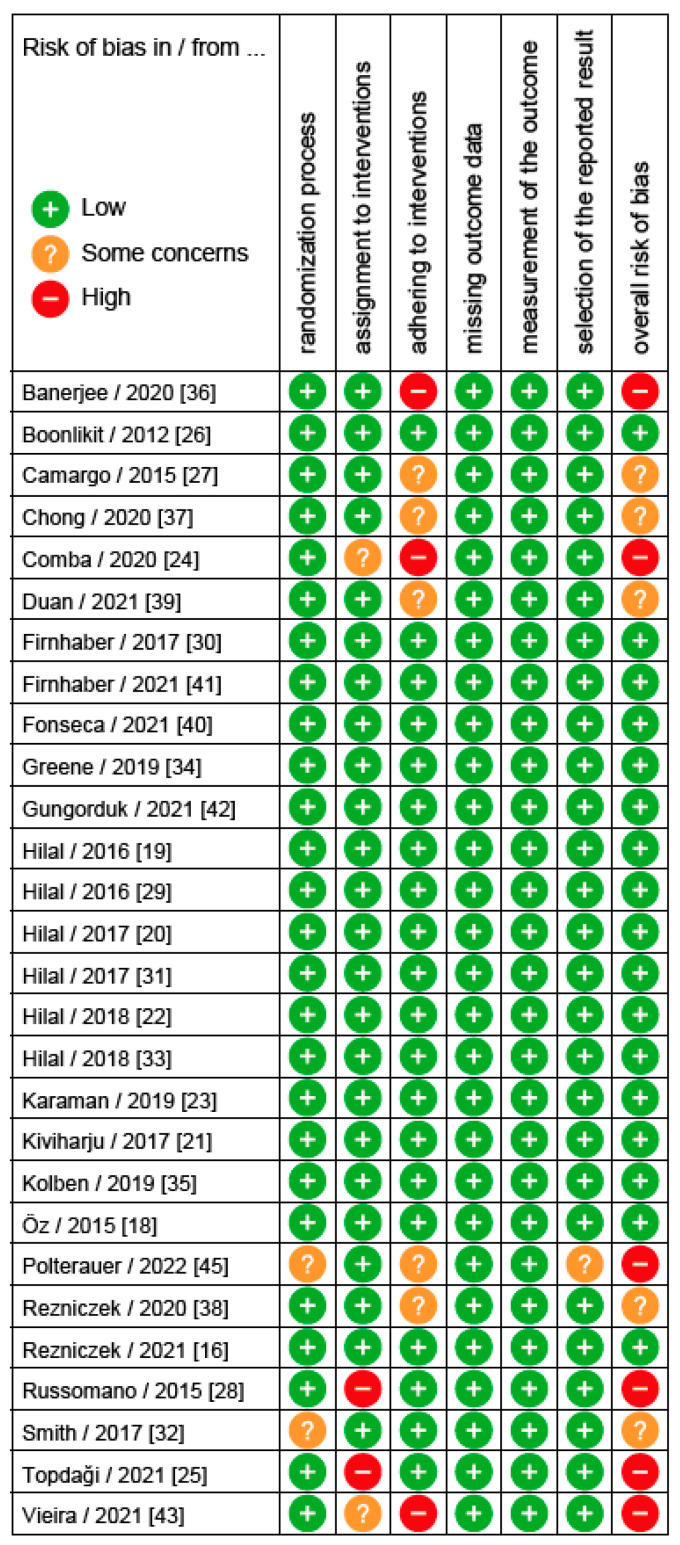
Methodological quality summary (according to the Cochrane Risk of Bias 2 tool [11]): Review authors’ judgements about each methodological quality item for each included study reporting on a randomized controlled trial (*n* = 28).

**Figure 3:**
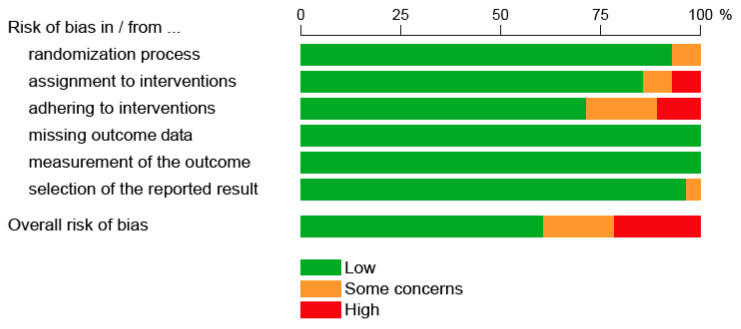
“Risk of bias” graph: Review authors’ judgements about each “Risk of bias” item presented as percentages across all included randomized controlled trials (*n* = 28). See Figure 2 for details.

**Figure 4:**
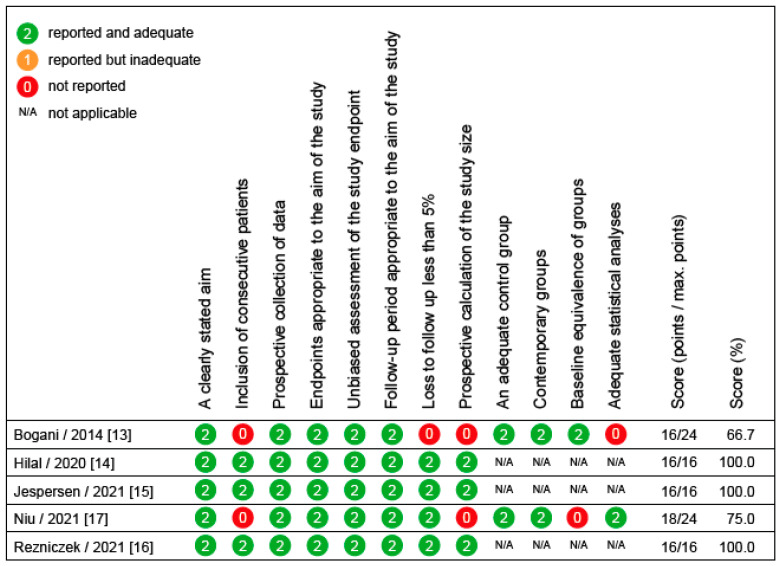
Methodological quality assessment and scores of prospective, non-randomized trials (*n* = 5), according to the revised methodological index for non-randomized studies (MINORS; [12]).

Figure 2 shows the methodological quality of all 21 RCTs. In 6/21 trials, there was a significant risk of bias [24,25,28,36,43,45], limiting the validity of the results. Figure 3 shows the overall and specific bias risks given as a percentage of all RCTs. This figure shows that assignment to and adherence to the study interventions were the main methodological problems causing a bad rating. In these cases, limiting the interpretation to the per protocol analyses might be useful. Figure 4 shows the methodological quality of the 5 diagnostic trials. In 2/5 trials, significant risks of bias were detected [13,17], limiting the validity of the results. Since both studies had multiple issues, the results of these studies must be interpreted with caution.

### 3.4. Systematic Reviews of Diagnostic or Therapeutic Interventions in Women with Cervical Dysplasia

We identified six systematic reviews and meta-analyses analyzing diagnostic or therapeutic interventions in women with cervical dysplasia [46,47,48,49,50,51]. Five of them analyzed therapeutic interventions. One systematic review compared different interventions to reduce blood loss during cervical surgery [46]. Two of the systematic reviews analyzed studies comparing cryotherapy and LEEP/LLETZ [48,50] and further two reviews analyzed photodynamic therapy, an experimental, non-invasive therapy of cervical dysplasia [49,51]. One systematic review looked at pain relief during colposcopy, the standard diagnostic intervention for women with suspected cervical dysplasia [47]. The specific details of the six systematic reviews are described below.

D’Alessandro et al. performed a meta-analysis of 4 trials with 1035 women with LSIL/HSIL and compared the efficacy of LEEP/LLETZ vs. cryotherapy [50]. Biopsy-proven LSIL/HSIL persistence after 6 m was the primary endpoint. LEEP/LLETZ was superior regarding the primary endpoint (relative risk [RR]: 0.87, 95% confidence interval [CI]: 0.76–0.99). The rate of biopsy-proven LSIL/HSIL after 12 m (secondary endpoint) also favored LEEP/LLETZ over cryotherapy (RR: 0.91, 95% CI: 0.84–0.99). Moreover, the superiority of LEEP/LLETZ was visible in the subgroups of women with HSIL only and HIV-positive women (RR: 0.89, 95% CI: 0.77–0.98 and RR: 0.88, 95% CI: 0.76–0.99, respectively). Complications did not differ between LEEP/LLETZ and cryotherapy.

Santesso et al. identified 167 randomized controlled trials and non-randomized controlled trials comparing three different types of surgery in women with LSIL/HSIL, i.e., LEEP/LLETZ, cold-knife conization, and cryotherapy [48]. They found that cold-knife conization was more effective compared to LEEP/LLETZ and cryotherapy but resulted in more short-term and long-term complications. Specifically, the rate of LSIL/HSIL recurrence 12 m after surgery was 5% for LEEP/LLETZ and cryotherapy compared to only 1.4% after cold-knife conization. On the other hand, there were fewer major bleeding episodes requiring hospital admission or blood transfusions after cryotherapy compared to cold-knife conization (RR 0.15; 95% CI 0.10–0.20) as well as fewer major infections (RR 0.17; 95% CI 0.07–0.43), fewer surgical complications (RR 0.11; 95% CI 0.03–0.38), and fewer episodes of minor bleeding (RR 0.03; 95% CI 0.02–0.06). Comparisons of cryotherapy and LEEP/LLETZ showed fewer infections (RR 0.12; 95% CI 0.06–0.28) and fewer episodes of minor bleeding (RR 0.46; 95% CI 0.37–0.56) after cryotherapy. The most important long-term complication after cervical surgery, premature birth, occurred most often after cold-knife conization (RR 3.41; 95% CI 2.38–4.88).

Pain relief during colposcopy was the focus of a systematic review and meta-analysis of 19 RCTs with 1720 probands by Gajjar et al. [47]. There was no difference in pain relief when using oral analgesics compared with placebo or no treatment (mean difference (MD) −3.51; 95% CI −10.03 to 3.01; 129 women), whereas the combination of an intracervical injection of a local anesthetic with a vasoconstrictor (e.g., lignocaine plus adrenaline or prilocaine plus felypressin) resulted in less pain (MD −23.73; 95% CI −37.53 to −9.93; 95 women).

Martin-Hirsch and Bryant analyzed RCTs aimed at assessing interventions to reduce blood loss during cervical surgery [46]. Twelve RCTs with 1520 probands were included in the meta-analysis. Vasopressin significantly reduced peri-operative bleeding (MD −100.80, 95% CI −129.48 to −72.12) and the risk of intra-operative bleeding (RR 0.39, 95% CI 0.27 to 0.56). Tranexamic acid also significantly reduced secondary bleeding episodes (RR 0.23, 95% CI 0.11 to 0.50) and post-operative blood loss (MD −55.60, 95% CI −94.91 to −16.29). Lastly, packing with Monsel’s solution reduced peri-operative blood loss (MD −22.00, 95% CI −23.09 to −20.91) and post-operative dysmenorrhea (RR 0.37, 95% CI 0.16 to 0.84) as well as unsatisfactory colposcopy (RR 0.43, 95% CI 0.30 to 0.63) and cervical stenosis (RR 0.35, 95% CI 0.25 to 0.49).

Zhang et al. analyzed the evidence regarding an alternative treatment to LEEP/LLETZ, namely photodynamic therapy, a non-invasive experimental local therapy of cervical dysplasia [49]. They included four RCTs with 433 probands. Compared with placebo, photodynamic therapy was safe and significantly increased the complete remission rate of LSIL/HSIL (odds ratio [OR] 2.51; 95% CI 1.23–5.12) as well as cervical HPV infection (OR 3.82; 95% CI 1.91–7.65). Specifically, the remission rates with photodynamic therapy were between 77% and 82%.

Unanyan et al. again analyzed the available evidence regarding photodynamic therapy 3 y later [51]. They identified six controlled trials and confirmed that photodynamic therapy was safe and more effective than placebo in the treatment of LSIL and HSIL and holds promise, particularly in young women, because it does not lead to obstetrical problems during subsequent pregnancies. However, no comparative trials vs. the standard therapy (LEEP/LLETZ) was identified.

### 3.5. Ongoing Studies

We searched the US government website clinicaltrials.gov to identify ongoing studies assessing diagnostic and therapeutic interventions in women with cervical dysplasia. Searching for “cervical dysplasia” resulted in 378 studies. Studies assessing cervical cancer screening strategies/interventions and those not evaluating diagnostic or therapeutic interventions on a patient-specific level were excluded (*n* = 351). We identified 27 studies matching the inclusion criteria. Study design and study characteristics are presented in Table 3. Eight currently ongoing studies look at the value of different surgical techniques, two studies at methods to improve the diagnosis of cervical dysplasia, 13 studies assess various experimental therapeutics, among them trichloracetic acid, curcumin, estradiol, and pembrolizumab. One study looks at the therapeutic effect of a licensed HPV vaccine and three studies prospectively assess spontaneous regression rates of LSIL/HSIL.

## 4. Discussion

Cervical dysplasia is one of the most common disorders of the female genital tract affecting millions of women worldwide. The World Health Organization (WHO) estimates that 1% to 2% of women worldwide develop HSIL every year [48]. The annual prevalence of HSIL among women living with the Human Immunodeficiency Virus (HIV) is even higher, at 10%. Therefore, evidence-based diagnostic and therapeutic interventions for LSIL/HSIL are an important worldwide medical need. In order to clarify what progress has been made in this field during the last decade, we systematically searched the literature between 2012 and 2021 and identified 39 high-quality controlled trials, RCTs, and PCTs, reporting on diagnostic and therapeutic interventions in women with cervical dysplasia [13,14,15,16,17,18,19,20,21,22,23,24,25,26,27,28,29,30,31,32,33,34,35,36,37,38,39,40,41,42,43,44,45,46,47,48,49,50,51]. Among them, we found 5 PCTs [13,14,15,16,17] and 28 RCTs [18,19,20,21,22,23,24,25,26,27,28,29,30,31,32,33,34,35,36,37,38,39,40,41,42,43,44,45], describing in summary 5935 patients. In addition, we found 6 systematic reviews and meta-analyses [46,47,48,49,50,51]. This amount of clinical trials demonstrates that significant progress in the diagnosis and treatment of LSIL/HSIL has been made during the last decade allowing us to outline up-to-date recommendations for an evidence-based diagnosis and treatment of affected women.

The diagnostic and therapeutic studies included in this review have variable methodological qualities. Therefore, we have addressed this and have graded the risk of bias of all 21 RCTs and 5 diagnostic trials. Of note, in a third of all trials, significant methodological limitations regarding patient selection, randomization process, and interpretation were identified. Thus, further confirmatory trials are necessary to assess if the effects shown in these studies [13,17,24,25,28,36,43,45] are real. Another important issue in studies investigating colposcopy and colposcopically-guided cervical biopsies is the experience of the colposcopists included in these studies. Only a minority of the studies included in this review have addressed this issue [31,33,38]. In the absence of information regarding the experience of the colposcopists, it cannot be ruled out that some of the studies included in this review have a limited external validity and results may not be applicable to settings with a high proportion of novices or exclusively expert settings.

In one of the studies included in our review [15], it was found that random biopsies are effective for identifying CIN2+ lesions, although a policy of non-targeted biopsies for women referred for colposcopy at the lowest level of risk and a completely normal colposcopic impression are not generally recommended. We do not recommend implementing a general policy of random biopsies. However, the data in Jespersen’s study [15] suggest that under specific circumstances such as in women with cytology of LSIL or ASCUS and a normal colposcopic impression, random biopsies my result in a higher yield of CIN2+. On the other hand, no study included in our systematic review indicates that random biopsies are of any value when a minor or major change lesion is visible. Another issue is that the level of colposcopic expertise has not been incorporated in the Jespersen study. Thus, the usefulness of random biopsies may be limited to unexperienced colposcopists who find it harder to correctly identify acetowhite and/or iodine-negative cervical lesions. Although not proven in a clinical trial, it seems reasonable to advise young colposcopists to use biopsies more liberally than experienced colleagues do.

An up-to-date recommendation for diagnostic interventions in women with suspected cervical dysplasia is as follows: Based on seven RCTs [18,20,21,22,23,24,25] we recommend intravenous or intracervical lidocaine for pain reduction during colposcopically-directed cervical biopsies. We do not recommend topical lidocaine [18,23], music, or video colposcopy (with the patient watching the procedure) during colposcopy [20,22]. Monsel’s solution might be used to control bleeding after cervical biopsies [19]. The acetic acid test should be scored 1 min after the application of acetic acid [14] and should be followed by Lugol’s iodine test for an optimal yield of LSIL/HSIL [16]. In women with cytology of LSIL or ASCUS and a normal colposcopic impression, 4 random cervical biopsies are useful [15].

An up-to-date recommendation for therapeutic interventions in women with cervical dysplasia is as follows: LEEP/LLETZ remains the standard and techniques such as SWETZ, C-LETZ, and TCBEE are not superior [26,27,28,31]. LEEP/LLETZ should be performed under local anesthesia and with direct colposcopic vision [33]. Spray coagulation for intra-operative bleeding control during LEEP/LLETZ is faster but equally effective compared to forced coagulation [29]. Cryotherapy and thermoablation might be used in women with LSIL, especially in women with HIV infection [30,32,36,39], but LEEP/LLETZ remains the standard for HSIL [34]. Topical imiquimod applied to the cervix or in the vagina seems to be safe and efficacious but remains an experimental procedure [40,45].

We also took care to see what the future holds for the management of women with cervical dysplasia. Among the studies currently enlisted at clinicaltrials.gov, the most exciting aspects are whether or not trichloracetic acid and pembrolizumab, a very cheap and a very expensive drug, respectively, will be added to the standard armamentarium of treating cervical dysplasia. In addition, reliable data from prospective studies will become available deciding whether it is safe to manage women with LSIL/HSIL with surveillance and for how long. Finally, data from RCTs will answer the question whether intra-operative Lugol’s iodine test during LEEP/LLETZ is useful for the definition of optimal resection margins and whether a limited form of LEEP only resecting biopsy-proven lesions instead of the whole transformation zone is safe. In summary, data will become available within the next 3–5 y further refining and optimizing the surgical and non-surgical treatment of cervical dysplasia.

## 5. Future Research Needs

Research on the diagnosis and therapy of cervical dysplasia is in constant progress. To underline this, we identified 27 ongoing clinical trials addressing various unresolved issues. For example, eight ongoing studies will assess different surgical techniques, among them intra-operative iodine test during LEEP/LLETZ, virtual reality-assisted LEEP/LLETZ, partial resection of the cervix compared to full LLETZ, and thermal ablation. Clearly, improving cervical surgery is a major medical research need and should be a focus of further clinical trials based on the high number of women who have to undergo cervical procedures. Second, conservative therapies obviating the need for surgery all together should be another important research focus. It will be important to see if substances such as trichloracetic acid, curcumin, estradiol, or pembrolizumab can effectively treat cervical dysplasia and spare affected women the risk of adverse pregnancy outcomes associated with cervical surgery. Finally, the potential therapeutic efficacy of HPV vaccines and their potential to reduce the recurrence risk after complete therapy of cervical dysplasia are another important field of future research.

## 6. Conclusions

In conclusion, we found that tremendous progress has been made in the last decade regarding both diagnostic interventions as well as therapeutic interventions for women with cervical dysplasia. Based on >30 controlled clinical trials, we were able to formulate specific and evidence-based recommendations.

## Figures and Tables

**Table 1:** Clinical characteristics of studies reporting on diagnostic procedures in women with cervical dysplasia.

Author/Year	Clinical Trial Registration	Study Type	Sample Size	Objective	Primary Endpoint	Numerical Results	Main Conclusions
Bogani et al., 2014 [13]	None	PCT	100	To compare the effect of 2 mL of intracervical lidocaine 1% vs. forced coughing for pain control during colposcopically guided biopsy	Procedure-related pain; using a 100-mm visual analogue scale	No between-group differences were observed in terms of pain related to speculum insertion, biopsies and pain recorded after the procedure (*p* > 0.05)	Forced coughing should be preferred over local anesthesia
Öz et al., 2015 [18]	None	RCT	214	To compare the effectiveness of topical lidocaine spray vs. placebo for relieving pain during colposcopically guided biopsy and ECC	Pain level immediately after the cervical biopsy and ECC, measured using the Wong-Baker FACES Pain Rating Scale	Pain scores were similar; mean ± SD pain scores were 2.18 ± 1.7 in the lidocaine group and 2.31 ± 1.6 in the control group	Routine use of a lidocaine spray before cervical punch biopsy or ECC is not recommended
Hilal et al., 2016 [19]	NCT02486471	RCT	145	To estimate the efficacy and side effects of Monsel’s solution for hemostasis after colposcopically guided biopsy	Vaginal bleeding after 15 min measured by scoring a sanitary pad with a 5-level pictogram	Mean bleeding score after 15 min with Monsel’s solution was 1.2 ± 0.6 vs. 1.8 ± 1.0 without Monsel’s solution (*p* < 0.001)	Monsel’s solution significantly reduces bleeding
Hilal et al., 2017 [20]	NCT02697175	RCT	225	To test whether video colposcopy reduces anxiety among patients undergoing colposcopically guided biopsy	Reduction of situation-specific anxiety scores (∆S = S2−S1) measured before (S1) and after (S2) colposcopy, using the State-Trait Anxiety Inventory	The mean ∆S was −10.3 ± 11.3 SD in the video colposcopy group and −10.3 ± 11.0 SD in controls (*p* = 0.50)	Video colposcopy does not reduce anxiety
Kiviharju et al., 2017 [21]	SRCTN20548888	RCT	204	To compare the effect of an intracervical anesthetic vs. no intervention for pain control during colposcopically guided biopsy	Pain experienced during colposcopic examination, using a 10-cm visual analog scale	Mean VAS score for the local anesthetic was 2.7 vs. 3.5 in controls (*p* = 0.017; 95% CI = 0.1–1.5)	Injection of a local anesthetic reduces pain compared to no intervention during colposcopically guided biopsy
Hilal et al., 2018 [22]	NCT03005795	RCT	212	To test whether music by Mozart reduces anxiety among patients undergoing during colposcopically guided biopsy	Reduction of the situation-specific anxiety of women hearing Mozart’s Symphony No. 40 during colposcopy, measured before and after colposcopy using the State-Trait Anxiety Inventory	The mean anxiety reduction was −9.4 ± 10.8 SD in the music group and −9.0 ± 10.6 in controls (*p* = 0.40)	Mozart’s Symphony No. 40 does not reduce anxiety in women undergoing during colposcopically guided biopsy
Karaman et al., 2019 [23]	NCT03100565	RCT	86	To compare the effectiveness of a local lidocaine spray compared to forced coughing for pain control during colposcopically guided cervical biopsy	Differences in pain perceived at four different consecutive steps during colposcopically guided cervical biopsies, assessed by using a 10 cm visual analogue scale	The mean ± SD pain scores after biopsy were 3.25 ± 1.4 in the lidocaine spray group and 4.4 ± 1.3 in the forced coughing group (*p* < 0.05)	Lidocaine spray can be recommended for pain relief during colposcopically directed cervical biopsy
Comba et al., 2020 [24]	NCT03279666	RCT	228	To compare pain perception during colposcopy with/ without tenaculum and with/without intracervical lidocaine/adrenaline	Pain perception during colposcopy assessed using a linear visual analogue scale and biopsy specimen size measured in millimeters in 4 arms (with/without tenaculum and with/without intracervical lidocaine plus adrenaline)	Tenaculum use increased pain perception in the without analgesic group; no differences were noted when the local analgesic was used; size and number of biopsy specimens did not affect pain	Administration of an intracervical analgesic reduces pain when a tenaculum is used
Hilal et al., 2020 [14]	None	PCT	300	To define the optimal timing for the colposcopic assessment of acetowhite lesions	Most severe colposcopic lesion 1, 3, and 5 min after application of acetic acid, using a standardized colposcopy protocol	After 1 min, 290 of 300 patients (96.7%) were diagnosed with the most severe colposcopic lesion; this proportion did not improve after 3 min (290/300 [96.7%]) or after 5 min (233/264 [88.3%])	The best time to identify lesions is 1 min after the application of acetic acid; fading of acetowhite lesions is common and supports a recommendation of not prolonging colposcopy beyond 3 min
Jespersen et al., 2021 [15]	NCT04249856	PCT	173	To determine the yield of CIN2+ from one to four cervical biopsies in women with cytology of LSIL or ASCUS and a normal colposcopic impression	CIN2+ in women with cytology of LSIL or ASCUS and a normal colposcopic impression	Four biopsies significantly increases CIN2+ cases vs. one biopsy (11.0% and 22.0%, *p* = 0.006)	Four random cervical biopsies at the squamocolumnar junction should be performed in women with cytology of LSIL or ASCUS who had a normal colposcopic impression
Rezniczek et al., 2021 [16]	None	PCT	320	To assess the performance of Lugol’s iodine test to identify HSIL/LSIL	Sensitivity/specificity of most severe iodine-negative lesions for the detection of LSIL/HSIL	The sensitivity and specificity of most severe iodine-negative lesions for the detection of LSIL/HSIL was 81.4 (95%-CI 77.3–85.0)% and 29.5 (24.2–35.5)%, respectively	Lugol’s iodine showed moderate sensitivity and poor specificity, but it changed clinical management in 5% of cases when used in addition to acetic acid
Topdaği et al., 2021 [25]	None	RCT	76	To investigate the effectiveness of intravenous lidocaine use in pain management during colposcopic cervical biopsy and ECC	Pain levels after i.v. lidocaine vs. no intervention measured using visual analogue scale scores	Pain scores were significantly lower in the lidocaine group than in the control group (*p* < 0.001)	Intravenous lidocaine administration can be used as an alternative approach to reduce pain and increase operator and patient satisfaction during colposcopy-directed biopsy and ECC

Abbreviations: ASCUS, atypical squamous cells of undetermined significance; CI, confidence interval; CIN, cervical intraepithelial neoplasia; ECC, endocervical curettage; HSIL, high-grade squamous intraepithelial lesion; LSIL, low-grade squamous intraepithelial lesion; PCT, prospective controlled trial, RCT, randomized controlled trial; SD, standard deviation; and VAS, visual analog scale.

**Table 2:** Clinical characteristics of studies reporting on therapeutic procedures in women with cervical dysplasia.

Author/Year	Clinical Trial Registration	Study Type	Sample Size	Objective	Primary Endpoint	Numerical Results	Main Conclusions
Boonlikit et al., 2012 [26]	None	RCT	98	To compare LLETZ with C-LETZ in the surgical management of CIN	Fragmentation of the operative specimen	C-LETZ was more likely to result in a single pathologic specimen (76 vs. 29.16%, *p* < 0.001); the incidence of incomplete excision and complications were similar in both groups	C-LETZ results in a higher rate of a single pathologic specimen but removes more cervical tissue than LLETZ
Camargo et al., 2015 [27]	NCT00995020	RCT	103	To compare SWETZ and LLETZ, for the surgical management of CIN	Rate of free endocervical margins	42 women in the LLETZ-cone group had free endocervical margin vs. 43 women in the SWETZ group (relative risk 1.04, 95% CI 0.87–1.25; *p* = 0.64)	SWETZ and LLETZ were equal with no difference regarding endocervical margin involvement
Russomano et al., 2015 [28]	NCT01929993	RCT	164	To compare SWETZ and LLETZ in women with a type 3 transformation zone regarding incomplete excision and other surgical outcomes	Resection margin status	LLETZ resulted in a higher risk of compromised or damaged endocervical margins compared to SWETZ (RR 1.72, 95% CI: 1.14 to 2.6); absolute risk reduction 26.4%	This study showed a lower proportion of compromised or damaged endocervical surgical margin in specimens resulting from SWETZ in relation to LLETZ
Hilal et al., 2016 [29]	NCT02330471	RCT	151	To evaluate spray and forced coagulation to achieve local hemostasis in women undergoing LLETZ	Time to complete local hemostasis	Mean (SD) time to complete local hemostasis with forced and spray coagulation was 43.3 (38.5) and 28.9 (22.9) s (*p* < 0.001)	Spray coagulation is superior to forced coagulation in women undergoing LLETZ; Spray coagulation should be used as the standard approach
Firnhaber et al., 2017 [30]	NCT02250716	RCT	220	To compare cryotherapy vs. no treatment in HIV-infected women with LSIL	Progression to HSIL after 12 m	Cryotherapy reduced progression to HSIL: 2/99 (2%) in the cryotherapy arm and 15/103 (15%) in the no treatment arm (86% reduction; 95% CI: 41% to 97%; *p* = 0.002)	Treatment of cervical LSIL with cryotherapy decreased progression to HSIL among HIV-infected women especially if high-risk HPV positive
Hilal et al., 2017 [31]	NCT02515162	RCT	172	To compare two conization techniques, LLETZ and TCBEE	Resection margin status	No difference in involved margin status between LLETZ and TCBEE was observed (12/91 [13%] vs. 7/81 [9%]; *p* = 0.4). Specimen fragmentation and surgeon preference favored LLETZ	LLETZ and TCBEE are equally safe and efficacious, but specimen fragmentation and surgeon preference favor LLETZ
Smith et al., 2017 [32]	NCT01723956	RCT	166	To compare the efficacy of LEEP vs. cryotherapy for the treatment of HSIL in HIV-seropositive women	6- and 12-m cumulative incidence of CIN2+	Cumulative CIN2+ incidence was higher for cryotherapy (24.3%; 95% CI, 16.1–35.8) than LEEP at 6 m (10.8%; 95% CI, 5.7–19.8) (*p* = 0.02), although by 12 m, the difference was not significant (27.2%; 95% CI, 18.5–38.9 vs. 18.5%; 95% CI, 11.6–28.8, *p* = 0.2)	Although rates of cumulative CIN2+ were lower after LEEP than cryotherapy treatment at 6 m, both treatments were equally effective in reducing CIN2+ by >70% by 12 m
Hilal et al., 2018 [33]	NCT02910388	RCT	182	To assess the benefits of performing LEEP under colposcopic guidance vs. no colposcopy	Resected cone mass	Women undergoing LEEP under colposcopic vision had significantly smaller cone specimens vs. controls (weight: median 1.86 (interquartile range 1.20–2.72) vs. 2.37 (interquartile range 1.63–3.31) g, *p* = 0.006)	LEEP with intra-operative colposcopy leads to significantly smaller cone specimens without compromising margin status
Greene et al., 2019 [34]	NCT01298596	RCT	400	To evaluate whether cryotherapy or LEEP is a more effective treatment for HSIL in women with HIV	Disease recurrence defined as CIN2 or higher on cervical biopsy during a 24-m follow-up	After 2 y, 60 women (30%) randomized to cryotherapy had recurrent CIN2 or higher vs. 37 (19%) in the LEEP group (relative risk, 1.71 (95% CI, 1.12–2.65); risk difference, 7.9% (95% CI, 1.9%–14.0%); *p* = 0.01)	Treatment with LEEP compared with cryotherapy resulted in a significantly lower rate of CIN recurrence over 24 m in women with HIV
Kolben et al., 2019 [35]	DRKS00006169	RCT	100	To show noninferiority of a limited-excision (resection of the dysplastic lesion only) vs. classical LLETZ	Rate of negative HPV tests after 6 m; trial was prematurely terminated	Patients in the limited-excision group did not show a lower number of negative HPV-tests (78% (LLETZ)-80% (limited-excision) = −2%; 90% confidence interval = −15%–12%)	Limited-excision may be an option to reduce surgical extent of cervical surgery; the trial was not sufficiently powered after premature termination due to lack of recruitment
Banerjee et al., 2020 [36]	CTRI/2017/06/008731	RCT	286	To compare the safety, acceptability, and efficacy of thermal ablation vs. cryotherapy in a screen and treat setting for CIN1+	Intensity of pain experienced during the procedure	Significantly more women treated by cryotherapy (75.3%) had pain compared to thermal ablation (61.0%), although intensity was mild in most cases	Thermal ablation reduces pain vs. cryotherapy in women with CIN1+; cure rates were comparable
Chong et al., 2020 [37]	KCT0003696	RCT	62	To evaluate the efficacy and feasibility of using a chitosan tampon (Hemoblock^®^) in preventing hemorrhage and enhancing wound healing after LEEP	Vaginal bleeding 2 w after surgery; measured daily with a pictorial blood assessment chart	The bleeding count was significantly lower in the chitosan group vs. controls (21.37 ± 16.86 vs. 40.52 ± 16.55, *p* = 0.0014)	The use of chitosan tampons can reduce hemorrhage, vaginal discharge, abdominal pain, and impairment of daily living after LEEP
Rezniczek et al., 2020 [38]	NCT03494686	RCT	208	To compare LEEP under local anesthesia vs. general anesthesia	Patient satisfaction assessed on the day of surgery and 14 d thereafter, using a Likert scale (score 0–100) and a questionnaire	Patient satisfaction did not differ between the study groups directly after surgery (Likert scale 100 (90–100) vs. 100 (90–100); *p* = 0.077) and 14 d thereafter (Likert scale 100 (80–100) vs. 100 (90–100); *p* = 0.079)	LEEP under local anesthesia is equally well tolerated and offers patient-reported and procedure-related benefits over general anesthesia
Duan et al., 2021 [39]	None	RCT	149	To compare thermocoagulation and cryotherapy for treatment of HSIL	Cytology-negative rate and HPV negative rate at follow-up at 4 and 8 m	No difference between thermocoagulation and cryotherapy regarding HPV-negative rates (4/8 m: 72.5%/86.2% vs. 68.6%/80.6%) (all *p* > 0.05); the cytology-negative rate was similar at 4 m (79.7% vs. 78.9%, *p* > 0.05), but higher for thermocoagulation at 8 m (100% vs. 88.7%, *p* < 0.05)	Thermocoagulation was as effective and safe as cryotherapy and might be easily applied to treat HSIL
Fonseca et al., 2021 [40]	NCT03233412	RCT	90	To evaluate the histologic response rate of HSIL after topical application of a 5% imiquimod cream	Rate of histologic regression (to CIN1 or less) in LEEP specimens	Histologic regression was observed in 23 of 38 participants (61%) in the experimental group compared with 9 of 40 (23%) in the controls (*p* = 0.001)	Weekly topical treatment with imiquimod is effective in promoting regression of HSIL
Firnhaber et al., 2021 [41]	NCT01928225	RCT	180	To evaluate if HPV vaccination improves response to treatment of cervical HSIL in women with HIV	Cervical HSIL by histology or cytology 26 and 52 w after HPV vaccine or placebo	HSIL was similar in the vaccine and placebo groups (53% vs. 45%; relative risk, 1.18 (95% CI, 0.87–1.6); *p* = 0.29)	This study did not support HPV vaccination to prevent recurrent HSIL after LEEP in women with HIV
Gungorduk et al., 2021 [42]	NCT03952975	RCT	73	To determine whether treatment of LSIL/HSIL in the follicular phase or luteal phase of the menstrual cycle affects peri- and post-operative blood loss during LEEP	Median early post-operative blood loss	Blood loss was lower during the follicular phase than during the luteal phase (209.2 (67.7–468.6) vs. 289.0 (120.3–552.8) mL; *p* = 0.01)	Performing LEEP during the follicular phase of the menstrual cycle significantly reduces intra-operative blood loss, early post-operative blood loss, and late post-operative blood loss
Niu et al., 2021 [17]	None	PCT	297	To compare the efficacy of 5-aminolaevulinic acid photodynamic therapy (5-ALA PDT) and CO_2_ laser in the treatment of LSIL with high-risk HPV	Complete remission rates at 4–6 and 12 m	After 4–6 m, there was no significant difference between the two groups, but after 12 m, complete remission rates were higher in the 5-ALA PDT group	The effect of 5-ALA PDT is similar to CO_2_ laser at 4–6 m; the long-term efficacy of 5-ALA PDT appears better
Vieira et al., 2021 [43]	NCT02500966	RCT	240	To compare the role of a new endocervical device to prevent cervical stenosis after LEEP in patients with HSIL	Rate of cervical stenosis at 30 d and 3, 6, and 12 m after intervention	The rate of cervical stenosis inDUDA group was (4–7.3%), and in No DUDA group was (4.3–5.8%) (*p* = 0.5)	The rate of cervical stenosis after LEEP was not reduced by an endocervical device
Rezniczek et al., 2022 [44]	NCT04326049	RCT	218	To compare LLETZ using video colposcopy vs. a headlight	Resected cone mass	LLETZ-video colposcopy and LLETZ-headlight (109 women each) had comparable cone masses (1.57 [0.98–2.37] vs. 1.67 [1.15–2.46] grams; *p* = 0.454)	Intra-operative video colposcopy for LLETZ results in equal cone masses
Polterauer et al., 2022 [45]	NCT01283763	RCT	93	To establish non-inferiority of a 16-w, self-applied topical imiquimod therapy vs. LLETZ in patients with HSIL	Negative HPV high-risk test 6 m after the start of treatment	In the imiquimod group, negative HPV test after 6 m was observed in 22/51 (43.1%) vs. 27/42 (64.3%) patients in the LLETZ group (rate difference 21.2%-points, 95% two-sided CI: 0.8 to 39.1)	In women with HSIL, imiquimod treatment results in lower HPV clearance rates when compared to LLETZ; LLETZ remains the standard of care

Abbreviations: ALA, 5-aminolaevulinic acid; C-LETZ, contour-loop excision of the transformation zone; CI, confidence interval; CIN, cervical intraepithelial neoplasia; DUDA, uterine device to dilate the endocervical canal; HIV, human immunodeficiency virus; HPV, human papillomavirus; HSIL, high-grade squamous intraepithelial lesion; ITT, intention to treat; LEEP, loop electrosurgical excision procedure; LLETZ, large loop excision of the transformation zone; LSIL, low-grade squamous intraepithelial lesion; PCT, prospective controlled trial; PDT, photodynamic therapy; RCT, randomized controlled trial; RR, relative risk; SD, standard deviation; SWETZ, straight-wire excision of transformation zone; and TCBEE, true cone biopsy electrode excision.

**Table 3:** Study characteristics of ongoing clinical trials listed at clinicaltrials.gov.

Location	Title	NCT	Study Type	Sample Size	Study Population	Interventions	Primary Endpoint(s)
Germany, Ruhr University Bochum	Comparison of Two Surgical Approaches in the Treatment of Cervical Dysplasia: Complete Removal of the Transformation Zone (LLETZ) vs. Isolated Resection of the Colposcopically Visible Lesion (LEEP)	04772937	RCT	206	Women with a LSIL/HSIL undergoing cervical surgery	LLETZ vs. limited cervical resection of LSIL/HSIL only	Rate of involved resection margins
Germany, Ruhr University Bochum	Large Loop Excision of the Transformation Zone (LLETZ) with vs. without Intra-operative Application of Lugol’s Iodine in Women with Cervical Dysplasia: a Prospective Randomized Trial	05132114	RCT	216	Women with a LSIL/HSIL undergoing LLETZ	Intra-operative Application of Lugol’s Iodine solution to define resection borders vs. standard LLETZ without application of Lugol’s Iodine solution	Rate of involved resection margins
Germany, Ruhr University Bochum	Impact of a VR Headset on Pain Perception and Satisfaction During Colposcopic Workup of Cervical Precancerous Lesions: a Multicenter Randomized-controlled Trial	04751799	RCT	286	Women undergoing colposcopy for suspected LSIL/HSIL	Virtual reality device before or before and during colposcopy vs. standard colposcopy	Patient anxiety and satisfaction
Denmark, University of Aarhus	See and Treat in an Outpatient Setting in Women above 45 Y with Cervical Dysplasia	04298957	PCT	150	Women ≥45 y with a positive cervical screening test and a T2/T3 type transformation zone	See-and-treat cone biopsy	Prevalence of CIN2+ lesions
Italy, Azienda USL Toscana Nord Ovest	A Randomised, Double-Blind, Placebo-Controlled, Phase III Study to Investigate the Efficacy of Presurgical 9-valent HPV Vaccination in Women Treated with LEEP for CIN2+ and Initially Invasive Cervical Cancer	03848039	RCT	1220	Women with histologically proven CIN2+ to early invasive cervical cancer ≤1a1	HPV vaccination (Gardasil9^®^) prior to cervical surgery and 2 m thereafter vs. placebo	CIN recurrence 5 y after surgical treatment
Austria, University of Vienna	TRICIN: Prospective Study on the Efficacy of Single Topical Trichloroacetic Acid (TCA) 85% in the Treatment of CIN1/2	04400578	PCT	101	Women with histologically proven CIN1/2	A single topical intervention of Trichloroacetic Acid (TCA) 85% on the cervix	CIN remission rate 6 m after intervention; safety and efficacy
USA, Guided Therapeutics, Inc.	The Use of the LuViva Advanced Cervical Scan to Identify Women at High Risk for Cervical Neoplasia	04915495	PCT	400	Scheduled for colposcopy for suspected LSIL/HSIL	Standardized colposcopy protocol + additional cervical biopsies based on LuViva + random biopsies	Sensitivity and specificity of the experimental device for CIN2+
Germany, University of Tübingen	Treatment of Cervical Intraepithelial Neoplasia (CIN) Grade III with Non-invasive Physical Plasma	04753073	RCT	40	Women with histologically proven CIN3	Topical cervical treatment with low temperature physical plasma followed by LEEP within 8 w vs. LEEP	Rate of complete CIN3 remission at the time of LEEP
Denmark, University of Copenhagen	Improving Diagnostic in Cervical Dysplasia: A Randomized Study with Local Estrogen Prior to Colposcopy	05283421	RCT	150	Women scheduled for colposcopy	Vaginal application of estrogen 30 µg once a day for 14 d prior to colposcopy vs. placebo	Visibility of the squamo-columnar junction at colposcopy
USA, Yale University	Treatment of High-Grade Pre-Neoplastic Cervical Lesions (CIN2/3) Using a Novel “Prime and Pull” Strategy	02864147	RCT	138	Women with HPV-positive CIN2/3	9-valent HPV vaccination twice (baseline and after 8 w) vs. weekly topical imiquimod 6.25 mg vaginal suppository for 16 w vs. observation	Regression to CIN1 or less after 20–24 m
USA, University of California at Los Angeles	A Phase II Open-Label, Single Arm Pilot Study to Evaluate the Safety and Efficacy of Pembrolizumab for High-Grade Cervical Intraepithelial Neoplasia	04712851	PCT	25	Women with histologically proven CIN2/3	Pembrolizumab every 6 w for 24 w	Pathological response rate at 6 m
China, Shanxi Academy of Medical Sciences	A Randomized Controlled Trial Comparing Cure Rates of Cervical Intraepithelial Neoplasia Grade 2 and Higher (CIN2+) Treated with CO_2_-based Cryotherapy, CropPen, and Thermoablation (UH3)	03084081	RCT	1152	Women with histologically proven CIN2/3	One 5 min freezing therapy (Cryopen) vs. 60 s thermoablation at 100 °C (thermoablation) vs. standard (ablative CO_2_ laser)	Residual CIN2+ at 12 m
USA, Frantz Viral Therapeutics, Inc.	A Phase II Double Blind, Placebo-controlled, Randomized Trial of Artesunate Vaginal Inserts for the Treatment of Patients with Cervical Intraepithelial Neoplasia (CIN2/3)	04098744	RCT	78	Women with histologically proven CIN2/3	Artesunate vaginal inserts, 200 mg/d for three 5-d cycles	Histological regression after 15 w
China, Peking University	Comparison of Cervical Intraepithelial Neoplasia 2/3 Treatment Outcomes with a Portable LMIC-adapted Thermal Ablation Device vs. Gas-based Cryotherapy	03429582	RCT	1282	Women with histologically proven CIN2/3	Thermoablation (cone tip) vs. thermoablation (detachable probe) vs. standard (cryotherapy)	Residual CIN2+ at 12 m
Zambia, International Agency for Research on Cancer and University of North Carolina Global Project Zambia and Liger Medical Llc	Development, Field Testing and Evaluation of the Efficacy of a Hand-held, Portable and Affordable Thermo-coagulator to Prevent Cervical Cancer in Low- and Middle-income Countries	02956239	RCT	450	Women with suspected cervical dysplasia	Thermoablation vs. cryotherapy vs. standard (LEEP)	
China, Peking Union Medical College Hospital	A Double Blind, Prospective, Randomized, Placebo Controlled, Multi-center Phase 3 Study to Evaluate Efficacy and Safety of Cevira^®^ in Patients with Cervical Histologic High-grade Squamous Intraepithelial Lesions (HSIL)	03870113	RCT	384	Women with histologically proven HSIL	Cevira^®^ (topical ointment + a single-use, disposable, LED-based red light source with continuous photoactivation of 125 J/cm^2^ over 4.6 h)	Histological response rates after 6 m
Spain, Hospital de la Santa Creu i Sant Pau	Conservative Management of Patients Diagnosed with High-grade Squamous Intraepithelial Lesions (H-SIL) Who Have Pregnancy Intentions: a Prospective Observational Study	04783805	PCT	200	Women with histologically proven CIN2/3	Conservative management with regular follow-up every 4 m with colposcopy and cytology at each visit	CIN2/3 regression after 2 y
USA, Johns Hopkins University	A Phase I Efficacy and Safety Study of HPV16-specific Therapeutic DNA-vaccinia Vaccination in Combination with Topical Imiquimod, in Patients with HPV16+ High Grade Cervical Dysplasia (CIN3)	00788164	PCT	75	Women with HPV 16-positive CIN3	Dose escalation study of a TA-HPV vaccine; pNGVL4a-Sig/E7(detox)/HSP70 DNA vaccine intramuscularly in weeks 0 and 4 and TA-HPV vaccine IM in week 8 vs. topical imiquimod once in weeks 0, 4, and 8 vs. pNGVL4a-Sig/E7(detox)/HSP70 DNA vaccine and TA-HPV vaccine + imiquimod	Safety, tolerability, and feasibility
USA, Johns Hopkins University	A Phase I Open Label, Dose Escalation Clinical Trial Assessing the Safety, Tolerability, and Feasibility of pNGVL4aCRTE6E7L2 HPV DNA Vaccine Administration Via Intramuscular TriGrid™ Electroporation Delivery System to Patients with HPV16-Positive High-Grade Cervical Intraepithelial Neoplasia	04131413	PCT	48	Women with HPV 16-positive CIN2 or HPV 16-positive CIN3	Dose escalation of an experimental vaccine, pNGVL4aCRTE6E7L2 with three escalating doses; Level 1 dose will be 0.3 mg	Dose-limiting toxicity
Sweden, University of Gothenburg	Expectancy as Alternative to Treatment for Cervical Intraepithelial Neoplasia Grade 2 Among Women 25–30 Y of Age. A Multicenter Clinical Study	03177863	PCT	160	Women with histologically proven CIN2	Expectant management with clinical visits every 6 m	Rate of regression after 24 m
France, University of Bordeaux	Therapeutic Abstention and Surveillance of Intra-epithelial Histological Lesions of High Grade Cervical CIN2 (Cervical Intraepithelial Neoplasia Grade 2). SUIVICIN	04057924	PCT	100	Women with histologically proven CIN2	Expectant management for 24 m	Rate of regression after 24 m
Israel, Tel Aviv Sourasky Medical Center	Virtual Reality During Conization of Cervix Uterus Under Local Anesthesia	04742543	RCT	100	Women undergoing cervical conization for dysplasia	Performance of conization with the use of virtual reality glasses vs. standard	Pain assessed by a defined score
USA, Emory University	An Investigation in the Use of Curcumin Topical Herbal Agent for the Treatment of Cervical Intraepithelial Neoplasia	04266275	RCT	200	Women with LSIL or recently treated HSIL	2000 mg of intravaginal curcumin once a week for 20 w vs. placebo	HPV clearance after 6 m
USA, University of Southern California	A Two-Cohort Randomized Phase 2 Trial of the IRX-2 Regimen in Women with Squamous Cervical Intraepithelial Neoplasia 3 (CIN3) or Vulvar Intraepithelial Neoplasia 3 (VIN 3)	03267680	RCT	60	Women with histologically confirmed CIN3 or usual type VIN 3	Cyclophosphamide IV on day 1 and IRX-2 via submucosal injections in the cervix or SC for vulvar lesions on days 4–7 plus indomethacin, multivitamins and omeprazole every 6 w for up to 2 courses	Pathological complete or partial remission after 25 w
Cuba, Our Lady of Rule No. 52 hospital	Evaluation of the Effect of the Combination of the Natural Products Glizigen^®^ and Ocoxin^®^-Viusid^®^ in the Treatment of High-grade Cervical Intraepithelial Lesions (Phase II)	03549273	PCT	62	Women with colposcopically diagnosed major change and HPV hr-positivity	Glizigen^®^ spray, topical use, 2 times a day for 6 m with an interruption for 2 m at the end of the third month and oral Ocoxin^®^-Viusid^®^ 60 mL daily for 8 m	Lesion progression on colposcopy after 9 m
China, Huazhong University of Science and Technology	Safety Study of Transcription Activator-like Effector Nucleases T512 in HPV16-infected Subjects	03226470	PCT	40	Women with HPV 16-infection	Biological T512 suppository contain 500 µg of T512 and suppocire (TALEN-T512)	Safety during 6 m
France, Centre Hospitalier Régional d’Orléans	Papilocare^®^: Effects on Regression of Histologically Confirmed Cervical Intraepithelial Lesions 1 and Tolerance	04624568	RCT	90	Women with histologically confirmed LSIL or ASC-US or LSIL cervical-cytology	Papilocare^®^ (hyaluronic acid and pre-biotics—Coriolus Versicolor—for 6 m with a single dose per day for 21 d over 28 during the first month, then 1 d over 2 during the following 5 m	Cervical cytology normalization after 12 m

Abbreviations: CI, confidence interval; CIN, cervical intraepithelial neoplasia; HPV, human papillomavirus; HSIL, high-grade squamous intraepithelial lesion; LEEP, loop electrosurgical excision procedure; LLETZ, large loop excision of the transformation zone; LSIL, low-grade squamous intraepithelial lesion; PCT, prospective controlled trial; and RCT, randomized controlled trial.

## Data Availability

The data presented in this study are available from the corresponding author upon reasonable request.

## Abbreviations

ASCUS, atypical squamous cells of undetermined significance; C-LETZ, contour-loop excision of the transformation zone; CI, confidence interval; CIN, cervical intraepithelial neoplasia; HPV, Human Papilloma Virus; HSIL, high-grade squamous intraepithelial lesions; LEEP, large loop excision of the transformation zone; LLETZ, large loop excision of the transformation zone; LSIL, low-grade squamous intraepithelial lesions; MD, mean difference; OR, odds ratio; PCT, non-randomized controlled trial; PICO, Population/Problem-Intervention/Exposure-Comparison-Outcome; PRISMA, Preferred Reporting Items for Systematic Reviews and Meta-Analyses; RCT, randomized controlled trial; RR, relative risk; SWETZ, Straight Wire Excision of the Transformation Zone; TCBEE, True Cone Biopsy Electrode Excision; and US, United States.
